# Classification of age-related macular degeneration using convolutional-neural-network-based transfer learning

**DOI:** 10.1186/s12859-021-04001-1

**Published:** 2021-11-08

**Authors:** Yao-Mei Chen, Wei-Tai Huang, Wen-Hsien Ho, Jinn-Tsong Tsai

**Affiliations:** 1 School of Nursing, Kaohsiung Medical University, Kaohsiung, 807 Taiwan; 2 Superintendent Office, Kaohsiung Medical University Hospital, Kaohsiung, 807 Taiwan; 3 Department of Mechanical Engineering, National Pingtung University of Science and Technology, Pingtung, 912 Taiwan; 4 Department of Healthcare Administration and Medical Informatics, Kaohsiung Medical University, Kaohsiung, 807 Taiwan; 5 Department of Medical Research, Kaohsiung Medical University Hospital, Kaohsiung, 807 Taiwan; 6 Department of Computer Science, National Pingtung University, Pingtung, 900 Taiwan

**Keywords:** Convolutional neural network, Transfer learning, Hyperparameter, Optical coherence tomography image, Age-related macular degeneration

## Abstract

**Background:**

To diagnose key pathologies of age-related macular degeneration (AMD) and diabetic macular edema (DME) quickly and accurately, researchers attempted to develop effective artificial intelligence methods by using medical images.

**Results:**

A convolutional neural network (CNN) with transfer learning capability is proposed and appropriate hyperparameters are selected for classifying optical coherence tomography (OCT) images of AMD and DME. To perform transfer learning, a pre-trained CNN model is used as the starting point for a new CNN model for solving related problems. The hyperparameters (parameters that have set values before the learning process begins) in this study were algorithm hyperparameters that affect learning speed and quality. During training, different CNN-based models require different algorithm hyperparameters (e.g., optimizer, learning rate, and mini-batch size). Experiments showed that, after transfer learning, the CNN models (8-layer Alexnet, 22-layer Googlenet, 16-layer VGG, 19-layer VGG, 18-layer Resnet, 50-layer Resnet, and a 101-layer Resnet) successfully classified OCT images of AMD and DME.

**Conclusions:**

The experimental results further showed that, after transfer learning, the VGG19, Resnet101, and Resnet50 models with appropriate algorithm hyperparameters had excellent capability and performance in classifying OCT images of AMD and DME.

## Background

Each year, almost 10 million individuals in the United States suffer from macular degeneration, also known as age-related macular degeneration (AMD), and more than 200,000 people develop choroidal neovascularization, a severe blinding form of advanced AMD [1, 2]. Additionally, nearly 750,000 individuals aged 40 or older suffer from diabetic macular edema (DME) [3], a vision-threatening form of diabetic retinopathy that causes fluid accumulation in the central retina. Many researchers have attempted to develop effective artificial intelligence algorithms by using medical images to diagnose key pathologies of AMD and DME quickly and accurately.

Naz et al. [4] addressed the problem of automatically classifying optical coherence tomography (OCT) images to identify DME. They proposed a practical and relatively simple approach to using OCT image information and coherent tensors for robust classification of DME. The features extracted from thickness profiles and cysts were tested using 55 diseased and 53 normal OCT scans in the Duke Dataset. Comparisons revealed that the support vector machine with leave-one-out had the highest accuracy of 79.65%. For identifying DME, however, acceptable accuracy (78.7%) was achieved by using a simple threshold based on the variation in OCT layer thickness. Najeeb et al. [5] used a computationally inexpensive single layer convolutional neural network (CNN) structure to classify retinal abnormalities in retinal OCT scans. After training using an open-source retinal OCT dataset containing 83,484 images from patients, the model achieved acceptable classification accuracy. In a multi-class comparison (choroidal neovascularization (CNV), DME, Drusen, and Normal), the model achieved 95.66% accuracy. Nugroho [6] used various methods, including histogram of oriented gradient (HOG), local binary pattern (LBP), DenseNet-169, and ResNet-50, to extract features from OCT images and compared the effectiveness of handcrafted and deep neural network features. The evaluated dataset contained 32,339 instances distributed in four classes (CNV, DME, Drusen, and Normal). The accuracy values for the deep neural network-based methods (88% and 89% for DenseNet-169 and ResNet-50, respectively) were superior to those for the non-automatic feature models (50% and 42% for HOG and LBP, respectively). The deep neural network-based methods also obtained better results in the underrepresented class. In Kermany et al. [7], a diagnostic tool based on a deep-learning framework was used to screen patients with common treatable blinding retinal diseases. By using transfer learning, the deep-learning framework could train a neural network with a fraction of the data required in conventional approaches. When an OCT image dataset was used to train the neural network, accuracy in classifying AMD and DME was comparable to that of human experts. In a multi-class comparison among CNV, DME, Drusen, and Normal, the framework achieved 96.1% accuracy. In Perdomo et al. [8], an OCT-NET model based on CNN was used for automatically classifying OCT volumes. The OCT-NET model was evaluated using a dataset of OCT volumes for DME diagnosis using a leave-one-out cross-validation strategy. Accuracy, sensitivity, and specificity all equaled 93.75%. The above results of research in AMD indicate that automatic classification accuracy needs further improvement.

Therefore, the motivation of this study was to find CNN-based models and their appropriate hyperparameters that use transfer learning to classify OCT images of AMD and DME. The CNN-based models were used for transfer learning included an 8-layer Alexnet model [9], a 22-layer Googlenet model [10], 16- and 19-layer VGG models (VGG16 and VGG19, respectively; [11]), and 18-, 50- and 101-layer Resnet models (Resnet18, Resnet50, and Resnet101, respectively; [12]). The algorithm hyperparameters included optimizer, mini-batch size, max-epochs, and initial learning rate. The experiments showed that, after transfer learning, the VGG19, Resnet101, and Resnet50 models with their appropriate algorithm hyperparameters had excellent performance and capability in classifying OCT images of AMD and DME.

This paper is organized as follows. The research problem is described in Sect. 2. Section 3 describes the research methods and steps. Section 4 presents and discusses the results of experiments performed to evaluate performance in classifying OCT images of AMD and DME. Finally, Sect. 5 concludes the study.

## Problem description

### AMD and DME

The macula, which is located in the center of the retina, is essential for clear visualization of nearby objects such as faces and text. Various eye problems can degrade the macula and, if left untreated, can even cause loss of vision. Age-related macular degeneration is a medical condition that can cause blurred vision or loss of vision in the center of the visual field. Early stages of AMD are often asymptomatic. Over time, however, gradual loss of vision in one or both eyes may occur. Loss of central vision does not cause complete blindness but can impair performance of daily life activities such as recognizing faces, driving, and reading. Macular degeneration typically occurs in older people. The classifications of AMD are early, intermediate, and late. The late type is further classified as “dry” and “wet” [13]. In the “dry” type, which comprises 90% of AMD cases, retinal deterioration is associated with formation of small yellow deposits, known as Drusen, under the macula. In the “wet” AMD type, abnormal blood vessel growth (i.e., CNV) occurs under the retina and macula. Bleeding and fluid leakage from these new blood vessels can then cause the macula to bulge or lift up from its normally flat position, thus distorting or destroying central vision. Under these circumstances, vision loss may be rapid and severe. A DME is characterized by breakdown of blood vessel walls in the retina resulting in accumulation of fluid and proteins in the retina. The resulting distortion of the macula then causes visual impairment or loss of visual acuity. One precursor of DME is diabetic retinopathy, in which blood vessel damage in the retina causes visual impairment [5].

### OCT images of AMD and DME

**Fig. 1:**
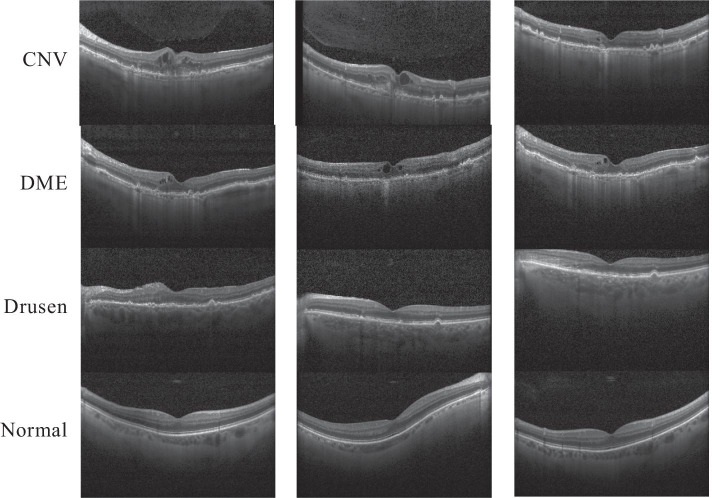
Representative optical coherence tomography images of the CNV, DME, Drusen, and Normal classes. *CNV* choroidal neovascularization, *DME* diabetic macular edema

In this study, all OCT images of AMD and DME used in the experiments were obtained from Kermany et al. [14]. The images were divided into four classes: CNV, DME, Drusen, and Normal. Figure 1 shows representative images of the four OCT classes.

### Considered problem

The considered problem was how to classify large numbers of different OCT images of CNV, DME, Drusen, and Normal efficiently and accurately. Since OCT images of CNV, DME, Drusen, and Normal can differ even for the same illness, a specialist or machine learning is needed to assist the physician in classifying the images.

## Methods

The research methods and steps were collecting data, processing OCT images of AMD and DME, selecting a pre-trained network for transfer learning, classifying OCT images of AMD and DME by CNN-based transfer learning, comparing performance among different CNN-based transfer learning approaches, and comparing performance with other approaches in classifying OCT images of AMD and DME. The detailed steps were as follows.

### Collecting data and processing OCT images of AMD and DME

The OCT images of AMD and DME in Kermany et al. [14] were split into a training set and a testing set of images. The training set had 83,484 images, including 37,205 CNV images, 11,348 DME images, 8,616 Drusen images, and 26,315 images of a normal eye condition. The testing set used for network performance benchmarking contained 968 images, 242 images per class. To maintain compatibility with the CNN-based architecture, each OCT image was processed as a 224 × 224 × 3 image, where 3 is the number of color channels.

### Selecting pre-trained network for transfer learning

Transfer learning is a machine learning method in which a model developed for a task is reused as the starting point for a model developed for another task. In transfer learning, pre-trained models are used as the starting point for performing computer vision and natural language processing tasks. Transfer learning is widely used because it reduces the computation time, the computational resources, and the expertise needed to develop neural network models for solving these problems [15]. In his NeurIPS 2016 tutorial, Ng [16] highlighted the potential uses of transfer learning and predicted that, after supervised learning, transfer learning will be the next major commercial application of machine learning. In transfer learning, a pre-trained model is used to construct a predictive model. Thus, the first step is to select a pre-trained source model from available models. The pool of candidate models may include models developed by research institutions and trained using large and complex datasets. The second step is to reuse the model. The pre-trained model can then be used as the starting point for a model used to perform the second task of interest. This may involve using all or parts of the model, depending on the modeling technique used. The third step is to tune the model. Depending on the input–output pair data available for the task of interest, the user may consider further modification or refinement of the model.

The widely used commercial software program Matlab R2019 by MathWorks has been validated as effective for pre-training neural networks for deep learning. The starting point for learning a new task was pretraining, in which the image classification network was pretrained to extract powerful and informative features from natural images. Most pre-trained networks were trained with a subset of the ImageNet database [17] used in the ImageNet Large-Scale Visual Recognition Challenge [18]. After training on more than 1 million images, the networks could classify images into 1000 object categories, e.g., keyboard, coffee mug, pencil, and various animals. Transfer learning in a network with pre-training is typically much faster compared to a network without pre-training.

### Classifying OCT images of AMD and DME by CNN-based transfer learning

**Fig. 2:**
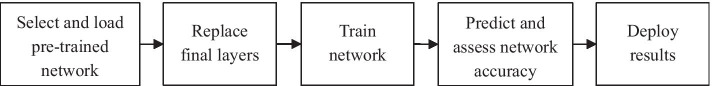
Flowchart of CNN-based transfer learning procedure

Fine-tuning a pre-trained CNN with transfer learning is often faster and easier than constructing and training a new CNN. Although a pre-trained CNN has already learned a rich set of image features, it can be fine-tuned to learn features specific to a new dataset, in this case, OCT images of AMD and DME. Fine-tuning a network is slower and requires more effort than simple feature extraction. However, since the network can learn to extract a different feature set, the final network is often more accurate. The starting point for fine tuning deeper layers of the pre-trained CNNs for transfer learning (i.e., Alexnet, Googlenet, VGG16, VGG19, Resnet18, Resnet50, and Resnet101) was training the networks with a new data set of OCT images of AMD and DME. Figure 2 is a flowchart of the CNN-based transfer learning procedure.

### Comparison of transfer learning performance in different CNN models

In the experiments, Alexnet, Googlenet, VGG16, VGG19, Resnet18, Resnet50, and Resnet101 were used to classify OCT images of AMD and DME in five independent runs. The results recorded for the training set and the testing set included (1) the accuracy in each run of the experiment, (2) the average accuracy for five independent runs, and (3) the standard deviation in the accuracy for five independent runs. Accuracy was defined as the proportion of true positive or true negative results for a population.

### Classification performance in comparison with other approaches

The accuracy, precision, recall (i.e., sensitivity), specificity, and F_1_-score values were used to compare performance with other approaches. Precision was assessed by positive predictive value (number of true positives over number of true positives plus number of false positives). Recall (sensitivity) was assessed by true positive rate (number of true positives over the number of true positives plus the number of false negatives). Specificity was measured by true negative rate (number of true negatives over the number of false positives plus the number of true negatives). The F_1_-score, a function of precision and recall, was used to measure prediction accuracy when classes were very imbalanced. In information retrieval, precision is a measure of the relevance of results while recall is a measure of the number of truly relevant results returned. The formula for F_1_-score is

1 $${\text{F}}_{1} {\text{ - score}} = 2 \times \frac{precision \times recall}{{precision + recall}}$$

## Results

The proposed CNN-based transfer learning method with appropriate hyperparameters was experimentally used to classify OCT images of AMD and DME. The OCT images in Kermany et al. [14] were used to train models and to test their performance. The experimental environment was Matlab R2019 and its toolboxes developed by The MathWorks. The network training options were the options available in the Matlab toolbox for CNN-based transfer learning with algorithm hyperparameters, i.e., ‘Optimizer’, ‘MiniBatchSize’, ‘MaxEpochs’ (maximum number of epochs), and ‘InitialLearnRate’.

The experimental data for OCT images of AMD and DME included a training set and a testing set. To maintain compatibility with the CNN-based architecture, each OCT image was processed as a 224 × 224 × 3 image, where 3 is the number of color channels. Table 1 shows the training and testing sets of OCT images of AMD and DME.

**Table 1:** Training and testing set of OCT images of AMD and DME

Class	Training set	Testing set	Total images
Normal	26,315	242	26,557
CNV	37,205	242	37,447
DME	11,348	242	11,590
Drusen	8616	242	8858
Total images	83,484	968	84,452

For training, different CNN-based models require different algorithm hyperparameters. The hyperparameter values are set before the learning process begins. Table 2 shows the selected CNN-based models with algorithm hyperparameters. The training option was use of ‘sgdm’, a stochastic gradient descent with a momentum optimizer. MiniBatchSize used a mini-batch with 40 observations at each iteration. MaxEpochs set the maximum number of epochs for training. InitialLearnRate was an option for dropping the learning rate during training.

**Table 2:** Selected CNN-based models with algorithm hyperparameters

CNN-based model	Hyperparameters			
	Optimizer	MiniBatchSize	MaxEpochs	InitialLearnRate
Alexnet	sgdm	40	5	10^–4^
Googlenet	sgdm	40	5	10^–4^
VGG16	sgdm	40	3	10^–4^
VGG19	sgdm	40	3	10^–4^
Resnet18	sgdm	40	5	10^–4^
Resnet50	sgdm	40	5	10^–4^
Resnet101	sgdm	40	5	10^–4^

For each CNN-based model, Table 3 shows the accuracy in each experiment, the average accuracy for all experiments, and the standard deviation (SD) in accuracy in classifying OCT images of AMD and DME. Data are shown for five independent runs of the experiments performed in the training set and in the testing set.

Table 3 shows that the average accuracy in the testing set ranged from 0.9750 to 0.9942 when using the CNN-based models with appropriate hyperparameters for transfer learning. For the testing set, the VGG19, Resnet101, and Resnet50 models had average accuracies of 0.9942, 0.9919, and 0.9909, respectively, which were all very high (all exceeded 0.99). Moreover, the SDs in accuracy obtained by VGG19 and Resnet101 were all 0.0005. That is, the VGG19 and Resnet101 had the most robust performance in classifying OCT images of AMD and DME.

**Table 3:** Accuracy of CNN models and standard deviation (SD) for classification of OCT images of AMD and DME in five independent runs of the experiments

CNN-based model	Image set	Experiments						
		1	2	3	4	5	Average accuracy	SD
Alexnet	Training set	0.9521	0.9479	0.953	0.9545	0.9508	0.9517	0.0025
	Testing set	0.9576	0.9928	0.9752	0.969	0.9804	0.9750	0.0131
Googlenet	Training set	0.9527	0.9525	0.9536	0.9539	0.9527	0.9531	0.0006
	Testing set	0.9845	0.9855	0.9793	0.9855	0.9845	0.9839	0.0026
VGG16	Training set	0.9592	0.9602	0.9602	0.9573	0.9602	0.9594	0.0013
	Testing set	0.9773	0.9959	0.9959	0.9783	0.9959	0.9887	0.0099
VGG19	Training set	0.9618	0.9593	0.961	0.9593	0.961	0.9605	0.0011
	Testing set	0.9938	0.9938	0.9948	0.9938	0.9948	0.9942	0.0005
Resnet18	Training set	0.9521	0.9509	0.9513	0.9507	0.9508	0.9512	0.0006
	Testing set	0.9866	0.9886	0.9804	0.9866	0.9814	0.9847	0.0036
Resnet50	Training set	0.9565	0.9507	0.9568	0.9572	0.9568	0.9556	0.0028
	Testing set	0.9917	0.9907	0.9897	0.9907	0.9917	0.9909	0.0008
Resnet101	Training set	0.9592	0.9584	0.9595	0.9595	0.9587	0.9591	0.0005
	Testing set	0.9917	0.9917	0.9917	0.9917	0.9928	0.9919	0.0005

**Fig. 3:**
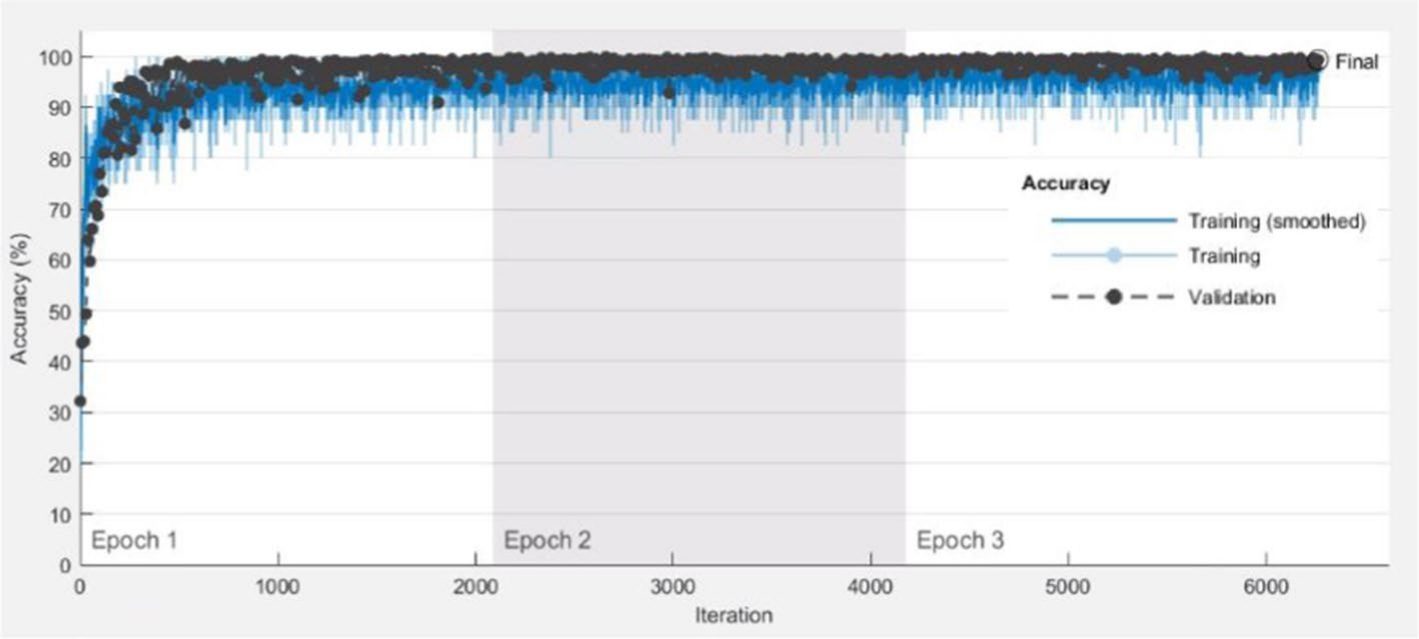
Progressive improvement in accuracy of VGG19

Figure 3 shows how model training progressively improved accuracy in VGG19. The selected training option was sgdm optimizer. MiniBatchSize used a mini-batch with 40 observations at each iteration. Iterations per epoch were 2087(= 83,484/40), which was the number of training images/MiniBatchSize. MaxEpochs, the maximum number of epochs, were set to 3. Therefore, maximum iterations were 6261(= 2087 × 3), which was iterations per epoch × MaxEpochs. The blue line shows the progressive improvement in accuracy for the training set, and the black line shows the progressive improvement in accuracy for the testing set.

**Fig. 4:**
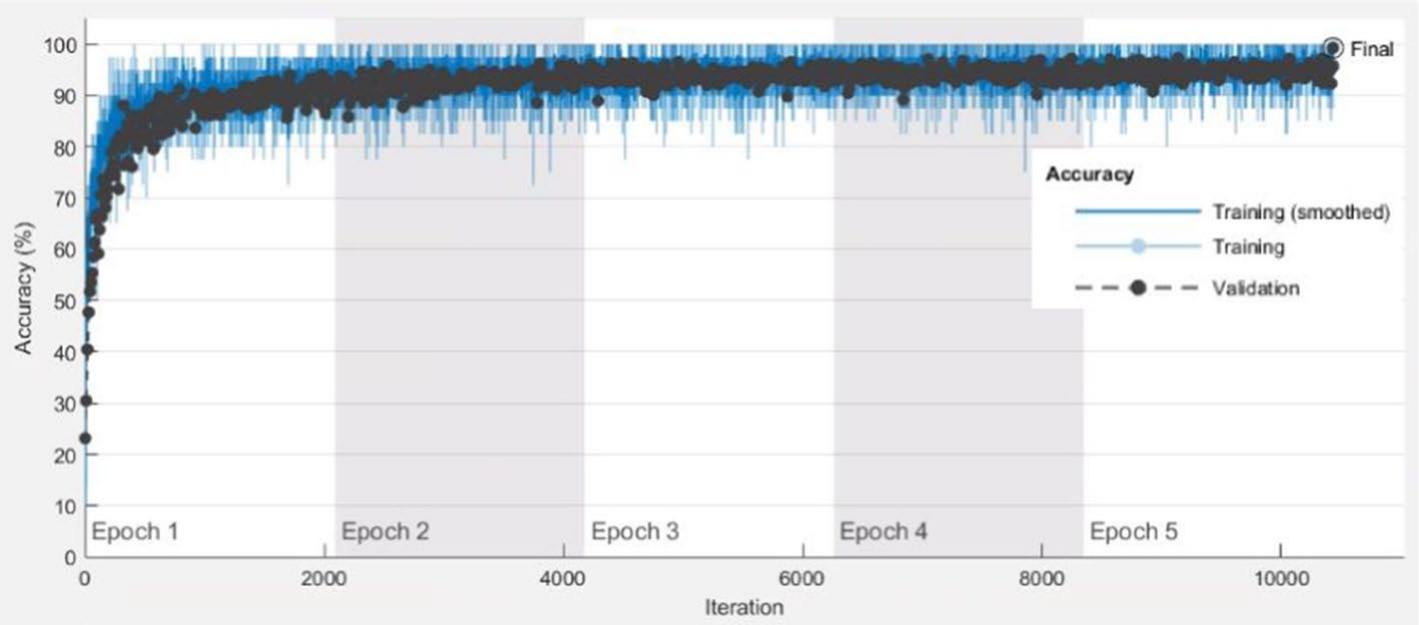
Progressive improvement in accuracy of Resnet101

**Fig. 5:**
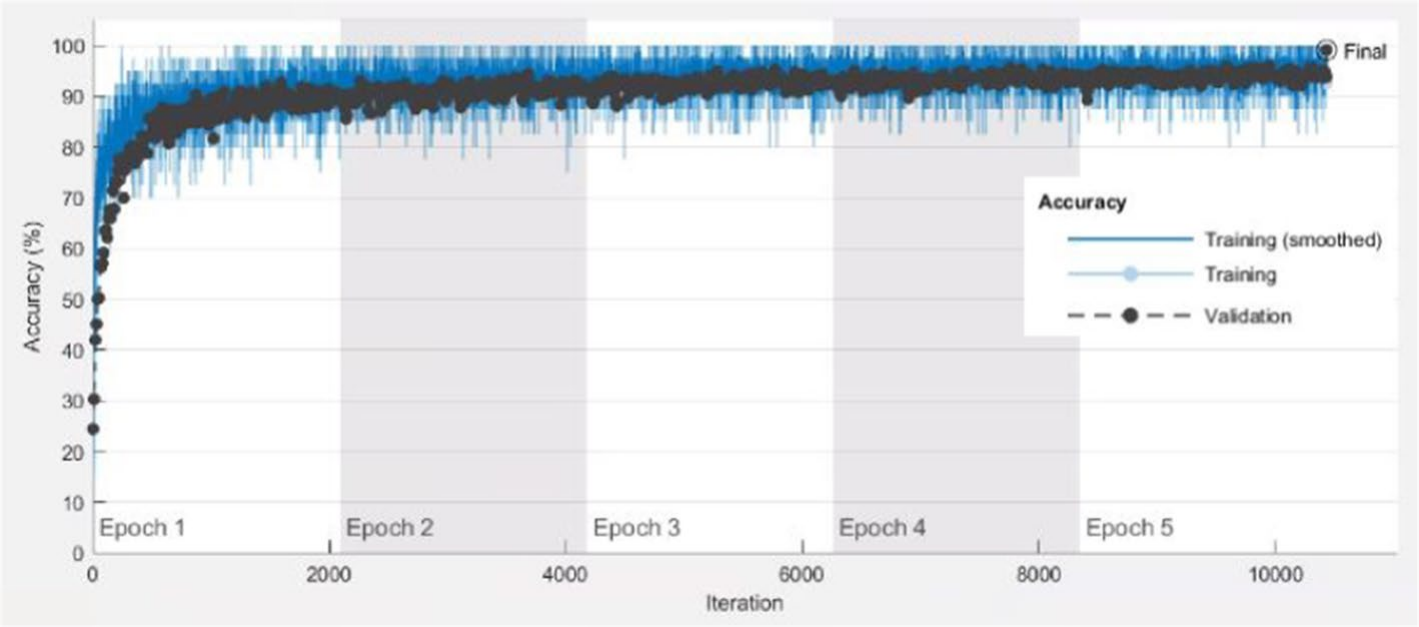
Progressive improvement in accuracy of Resnet50

Figures 4 and 5 show how model training progressively improved accuracy in Resnet101 and Resnet50, respectively. The training option was sgdm optimizer. MiniBatchSize used 40 observations at each iteration. Iterations per epoch were 2087. MaxEpochs were set to 5. Therefore, the maximum iterations were 10,435(= 2087 × 5). The blue line shows the progressive improvement in accuracy when using the training set, and the black line shows the progressive improvement in accuracy when using the testing set.

The accuracy metric was used to measure the transfer learning performance of the CNN-based models. Precision, recall, specificity, and F_1_-score were further used to validate classification performance. The results were depicted by creating a confusion matrix of the predicted labels versus the true labels for the respective disease classes. Tables 4, 5 and 6 show the confusion matrices used in multi-class comparisons of Normal, CNV, DME, and Drusen for VGG19, Resnet101, and Resnet50 for the testing data.

**Table 4:** Confusion matrix for Normal, CNV, DME, and Drusen obtained by VGG19 in Experiment #5

True Labels							
	Normal	CNV	DME	Drusen	Precision	F_1_-score	Average recall and specificity
Predicted labels							
Normal	242	0	0	0	1.0000	1.0000	
CNV	0	242	1	3	0.9837	0.9918	
DME	0	0	241	1	0.9959	0.9959	
Drusen	0	0	0	238	1.0000	0.9917	
Recall	1.0000	1.0000	0.9959	0.9835			0.9948
Specificity	1.0000	0.9945	0.9986	1.0000			0.9983
Average precision and F_1_-score					0.9949	0.9948	
Accuracy	0.9948

**Table 5:** Confusion matrix for Normal, CNV, DME, and Drusen obtained by Resnet101 in Experiment #5

True Labels							
	Normal	CNV	DME	Drusen	Precision	F_1_-score	Average recall and specificity
Predicted labels							
Normal	242	0	1	1	0.9918	0.9959	
CNV	0	241	2	2	0.9837	0.9897	
DME	0	1	239	0	0.9958	0.9917	
Drusen	0	0	0	239	1.0000	0.9938	
Recall	1.0000	0.9959	0.9876	0.9876			0.9928
Specificity	0.9972	0.9945	0.9986	1.0000			0.9976
Average precision and F_1_-score					0.9928	0.9928	
Accuracy	0.9928

**Table 6:** Confusion matrix for Normal, CNV, DME, and Drusen obtained by Resnet50 in Experiment #5

True labels							
	Normal	CNV	DME	Drusen	Precision	F_1_-score	Average recall and specificity
Predicted labels							
Normal	242	0	1	0	0.9959	0.9979	
CNV	0	240	1	4	0.9796	0.9856	
DME	0	2	240	0	0.9917	0.9917	
Drusen	0	0	0	238	1.0000	0.9917	
Recall	1.0000	0.9917	0.9917	0.9835			0.9917
Specificity	0.9986	0.9931	0.9972	1.0000			0.9972
Average precision and F_1_-score					0.9918	0.9917	
Accuracy	0.9917						

Table 4 shows that, in Experiment #5, VGG19 achieved an accuracy of 0.9948 with an average precision of 0.9949, an average recall of 0.9948, an average specificity of 0.9983, and an average F_1_-score of 0.9948.

Table 5 shows that, in Experiment #5, Resnet101 achieved an accuracy of 0.9928 with an average precision of 0.9928, an average recall of 0.9928, an average specificity of 0.9976, and an average F_1_-score of 0.9928.

Table 6 indicates that, in Experiment #5, Resnet50 achieved an accuracy of 0.9917 with an average precision of 0.9918, an average recall of 0.9917, an average specificity of 0.9972, and an average F_1_-score of 0.9917.

Next, the performance of the proposed CNN-based transfer learning approach in classifying OCT images of AMD and DME was compared with the results reported in Kermany et al. [7], Najeeb et al. [5], and Nugroho [6]. Table 7 shows the confusion matrix for Normal, CNV, DME, and Drusen obtained by Kermany et al. [7]. The model in Kermany et al. [7] achieved an accuracy of 0.9610 with an average precision of 0.9610, an average recall of 0.9613, an average specificity of 0.9870, and an average F_1_-score of 0.9610. Table 8 shows the confusion matrix for Normal, CNV, DME, and Drusen obtained by Najeeb et al. [5]. The model in Najeeb et al. [5] achieved an accuracy of 0.9566 with an average precision of 0.9592, an average recall of 0.9566, an average specificity of 0.9855, and an average F_1_-score of 0.9563.

**Table 7:** Confusion matrix for Normal, CNV, DME, and Drusen obtained by Kermany et al. [7]

True labels							
	Normal	CNV	DME	Drusen	Precision	F_1_-score	Average recall and specificity
Predicted labels							
Normal	246	0	0	4	0.9840	0.9762	
CNV	0	242	5	3	0.9680	0.9528	
DME	3	9	237	1	0.9480	0.9595	
Drusen	5	7	2	236	0.9440	0.9555	
Recall	0.9685	0.9380	0.9713	0.9672			0.9613
Specificity	0.9946	0.9892	0.9828	0.9815			0.9870
Average precision and F_1_-score					0.9610	0.9610	
Accuracy	0.9610

**Table 8:** Confusion matrix for normal, CNV, DME, and Drusen obtained by Najeeb et al. [5]

True labels							
	Normal	CNV	DME	Drusen	Precision	F_1_-score	Average recall and specificity
Predicted labels							
Normal	237	0	9	0	0.9634	0.9713	
CNV	0	241	20	5	0.9060	0.9488	
DME	0	0	211	0	1.0000	0.9316	
Drusen	5	1	2	237	0.9673	0.9733	
Recall	0.9793	0.9959	0.8719	0.9793			0.9566
Specificity	0.9876	0.9656	1.0000	0.9890			0.9855
Average precision and F_1_-score					0.9592	0.9563	
Accuracy	0.9566						

For the testing set, Table 9 shows the classifier accuracy, average precision,average recall/sensitivity, average specificity, and average F_1_-score obtained by the different CNN-based models. When the testing set was used in Experiment #5, the accuracies obtained by VGG19, Resnet101, and Resnet50 were 0.9948, 0.9928, and 0.9917, respectively, which are all very high and were superior to the accuracies obtained by the models in Kermany et al. [7], Najeeb et al. [5], and Nugroho [6]. In Experiment #5, other measures (i.e., average precision, average recall/sensitivity, average specificity, and average F_1_-score) obtained byVGG19, Resnet101, and Resnet50 were higher than those obtained by the models in Kermany et al. [7], Najeeb et al. [5], and Nugroho [6]. That is, by using transfer learning with appropriate hyperparameters, the proposed CNN-based models VGG19, Resnet101, and Resnet50 had excellent performance and capability in classifying OCT images of AMD and DME.

**Table 9:** Classifier accuracy, precision, recall/sensitivity, specificity, and F_1_-score obtained by different CNN-based models for testing set

CNN-based model	Accuracy	Average precision	Average recall/sensitivity	Average specificity	Average F_1_-score
VGG19-Experiment #5	0.9948	0.9949	0.9948	0.9983	0.9948
Resnet101-Experiment #5	0.9928	0.9928	0.9928	0.9976	0.9928
Resnet50-Experiment #5	0.9917	0.9918	0.9917	0.9972	0.9917
Kermany et al. [7]	0.9610	0.9610	0.9613	0.9870	0.9610
Najeeb et al. [5]	0.9566	0.9592	0.9566	0.9855	0.9563
Nugroho [6]-ResNet	0.8926	0.91	0.89	NA	0.89
Nugroho [6]-DenseNet	0.8802	0.90	0.88	NA	0.88

## Discussions

In this study, the appropriate algorithm hyperparameters for CNN-based transfer learning were very important for classifying OCT images of AMD and DME. This phenomenon was demonstrated by experiments in which the VGG19, Resnet50, and Resnet101 models achieved a classification accuracy exceeding 99%. If an inappropriate combination of algorithm hyperparameters is used, the classification accuracy will be reduced. For example, the algorithm hyperparameters for Googlenet transfer learning and the results in Table 10 indicates that an appropriate set of hyperparameters can provide good performance for transfer learning, where Optimizer of sgdm and InitialLearnRate of 10^–4^ are identical. Therefore, the combination of algorithm hyperparameters of the third case (i.e., Optimizer of sgdm, MiniBatchSize of 40, MaxEpochs of 5, and InitialLearnRate of 10^–4^) was selected for the study because it achieved high accuracy in the training and testing sets. Tables 11 and 12 show the algorithm hyperparameters for Resnet50 and Resnet101 transfer learning and their respective results. Tables 11 and 12 show that, if all other hyperparameter are identical (Optimizer of sgdm, MiniBatchSize of 40, and InitialLearnRate of 10^–4^), changing MaxEpochs from 3 to 5 improves accuracy for the test set by more than 0.99. Therefore, this combination of algorithm hyperparameters (i.e., Optimizer of sgdm, MiniBatchSize of 40, MaxEpochs of 5, and InitialLearnRate of 10^–4^) was selected for Resnet50 and Resnet101 transfer learning in classifying OCT images of AMD and DME.

**Table 10:** Googlenet model with different algorithm hyperparameters: accuracy for training and testing sets

Case number	Hyperparameters				Accuracy for training set	Accuracy for testing set
	Optimizer	MiniBatchSize	MaxEpochs	InitialLearnRate		
1	sgdm	80	5	10^–4^	0.9409	0.9680
2	sgdm	60	5	10^–4^	0.9495	0.9835
3	sgdm	40	5	10^–4^	0.9527	0.9845
4	sgdm	40	4	10^–4^	0.9472	0.9607
5	sgdm	20	3	10^–4^	0.9494	0.9452
6	sgdm	20	2	10^–4^	0.9480	0.9804

**Table 11:** Resnet50 model with different algorithm hyperparameters: accuracy for training and testing sets

Case number	Hyperparameters				Accuracy for training set	Accuracy for testing set
	Optimizer	MiniBatchSize	MaxEpochs	InitialLearnRate		
1	sgdm	40	3	10^–4^	0.9509	0.9824
2	sgdm	40	5	10^–4^	0.9568	0.9917

**Table 12:** Resnet101 model with different algorithm hyperparameters: accuracy for training and testing sets

Case number	Hyperparameters				Accuracy for training set	Accuracy for testing set
	Optimizer	MiniBatchSize	MaxEpochs	InitialLearnRate		
1	sgdm	40	3	10^–4^	0.9527	0.9876
2	sgdm	40	5	10^–4^	0.9587	0.9928

**Fig. 6:**
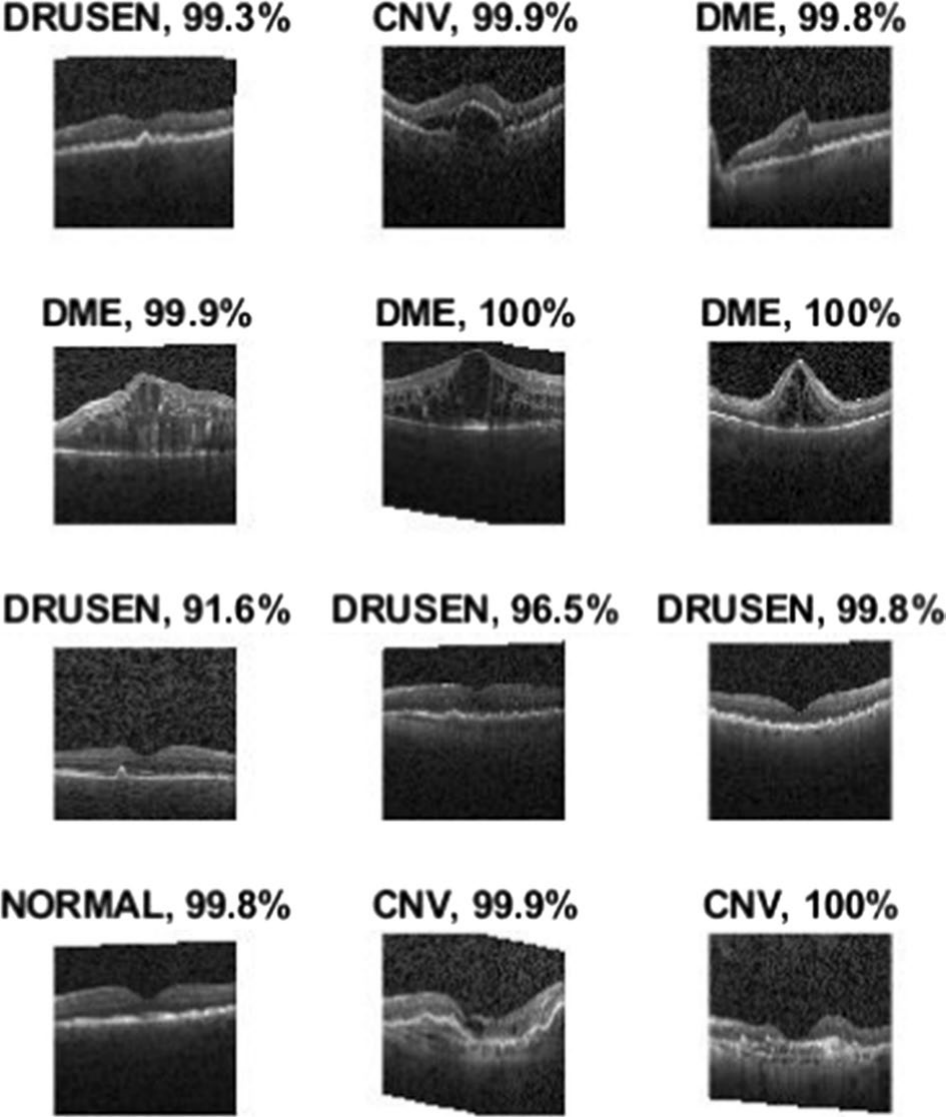
Four sample images with predicted labels and predicted probabilities of images with those labels

Figure 6 displays four sample images with predicted labels and the predicted probabilities of images with those labels. The results for four randomly selected sample images were very similar to the results for the predicted category, and the probabilities of prediction approached 100%, indicating that the model established by CNN-based transfer learning had high classification ability.

Presently, CNN-based transfer learning is very efficient and stable [19, 20]. The key to successful image classification is ensuring that the original images are correctly classified. This phenomenon was demonstrated by experiments in this study in which the CNN-based model achieved a classification accuracy exceeding 99%. Therefore, CNN-based transfer learning with appropriate hyperparameters has the best performance in classifying OCT images of AMD and DME.

## Conclusions

This study used CNN-based transfer learning with appropriate algorithm hyperparameters for effectively classifying OCT images of AMD and DME. The main contribution of this study is the confirmation that suitable CNN-based models with their algorithm hyperparameters can use transfer learning to classify OCT images of AMD and DME. Various metrics were used to verify the usability of the adopted CNN-based models. As Table 3 shows, the average accuracies of models VGG19, Resnet101, and Resnet50 in the testing set were 0.9942, 0.9919, and 0.9909, respectively, which were all very high (greater than 0.99). Moreover, the SDs of accuracy obtained by VGG19 and Resnet101 were all 0.0005. That is, VGG19 and Resnet101 were robust models for classifying OCT images of AMD and DME. Table 9 shows that, when the testing set was used in Experiment #5, the accuracies of VGG19, Resnet101, and Resnet50 were 0.9948, 0.9928, and 0.9917, respectively, which were all higher than the accuracies obtained by the models in Kermany et al. [7], Najeeb et al. [5], and Nugroho [6]. Other measures (average precision, average recall/sensitivity, average specificity, and average F_1_-score) obtained by VGG19, Resnet101, and Resnet50 in Experiment #5 were also higher than those obtained by the models in Kermany et al. [7], Najeeb et al. [5], and Nugroho [6]. That is, the CNN-based models VGG19, Resnet101, and Resnet50 obtained by transfer learning with appropriate algorithm hyperparameters were effective and useful for classifying OCT images of AMD and DME.

## Data Availability

All data information or analyzed during this study are included in this article.

## Abbreviations

CNV: Choroidal neovascularization
DME: Diabetic macular edema
AMD: Age-related macular degeneration
OCT: Optical coherence tomography
